# Resistance Training Improves Beta Cell Glucose Sensing and Survival in Diabetic Models

**DOI:** 10.3390/ijms23169427

**Published:** 2022-08-21

**Authors:** Gabriela Alves Bronczek, Gabriela Moreira Soares, Carine Marmentini, Antonio Carlos Boschero, José Maria Costa-Júnior

**Affiliations:** 1 Obesity and Comorbidities Research Center, Institute of Biology, University of Campinas (UNICAMP), Campinas 13083-864, Brazil; 2 Center for Diabetes Research, Division of Endocrinology, Erasmus Hospital, Universite Libre de Bruxelles (ULB), 1070 Brussels, Belgium

**Keywords:** diabetes, inflammation, glycemia, insulin, metabolism, health, exercise, streptozotocin

## Abstract

Resistance training increases insulin secretion and beta cell function in healthy mice. Here, we explored the effects of resistance training on beta cell glucose sensing and survival by using in vitro and in vivo diabetic models. A pancreatic beta cell line (INS-1E), incubated with serum from trained mice, displayed increased insulin secretion, which could be linked with increased expression of glucose transporter 2 (GLUT2) and glucokinase (GCK). When cells were exposed to pro-inflammatory cytokines (in vitro type 1 diabetes), trained serum preserved both insulin secretion and GCK expression, reduced expression of proteins related to apoptotic pathways, and also protected cells from cytokine-induced apoptosis. Using 8-week-old C57BL/6 mice, turned diabetic by multiple low doses of streptozotocin, we observed that resistance training increased muscle mass and fat deposition, reduced fasting and fed glycemia, and improved glucose tolerance. These findings may be explained by the increased fasting and fed insulinemia, along with increased beta cell mass and beta cell number per islet, observed in diabetic-trained mice compared to diabetic sedentary mice. In conclusion, we believe that resistance training stimulates the release of humoral factors which can turn beta cells more resistant to harmful conditions and improve their response to a glucose stimulus.

## 1. Introduction

Type 1 diabetes (T1D) is an autoimmune disease in which a subclass of T lymphocytes induces apoptosis of pancreatic beta cells, leading to insulin deficiency [1,2]. The absence of insulin promotes hyperglycemia, which results not only in micro- and macrovascular complications [3], but also affects the immune system itself. Hyperglycemia caused by diabetes leads to chronic exposure of immune cells to high glucose levels, generating alterations in intracellular metabolic pathways in innate and adaptive cells with subsequent immune hyperactivation [4].

Exercise is linked to reduced inflammation [5,6] and cardiovascular risk factors, weight loss, improved overall physical and mental wellbeing, as well as improved glucose homeostasis [7]. Endurance exercise, which has been extensively studied in the past years, improves glucose homeostasis, as well as beta cell function and survival in diabetic conditions [8,9,10,11,12,13,14,15]. However, this type of exercise conveys a high risk of hypoglycemia in T1D patients [16,17]. In contrast, resistance exercise, which has been less explored, reduces fasting glucose, insulin, and glycated hemoglobin (HbA1c), and improves beta cell function in diabetic patients, while representing a lower risk of hypoglycemic events [10,16,17,18,19,20]. Resistance-trained mice present reduced glucose levels during a glucose challenge due to the increased capacity of beta cells to secrete insulin. Furthermore, serum from resistance-trained mice reduces beta cell injury and apoptosis in a rat pancreatic beta cell line (INS-1E) exposed to a chemical endoplasmic reticulum (ER) stressor [21].

Since T1D patients present progressive loss of beta cells leading to insulin deficiency and altered glucose metabolism [3,22], the search for approaches such as resistance exercise, that could benefit insulin-producing cells, is of great interest. Therefore, this is one of the first studies investigating if resistance training could improve glucose metabolism on beta cells and protect these cells from apoptosis in an in vitro model of type 1 diabetes induced by pro-inflammatory cytokines, as well as in streptozotocin-induced diabetic (MLDS) mice.

Here, we demonstrate that the serum from healthy resistance-trained mice improves glucose sensing by increasing the expression of glucose transporter 2 (GLUT2) and glucokinase (GCK), and also reduces injury and apoptosis in INS-1E beta cells exposed to pro-inflammatory cytokines. In addition, diabetic-trained mice display improved glucose tolerance, reduced fasting and fed glycemia, along with increased insulinemia. Resistance training also promoted an increase in beta cell mass and beta cell number per islet in MLDS mice. These findings suggest that resistance training could be an important strategy to protect beta cells from impaired functioning and apoptosis in the diabetes context.

## 2. Results

### 2.1. Serum from Resistance-Trained Mice Improves Glucose Sensing and Preserves Insulin Secretion in INS-1E Cells Exposed to Pro-Inflammatory Cytokines

**Figure 1:**
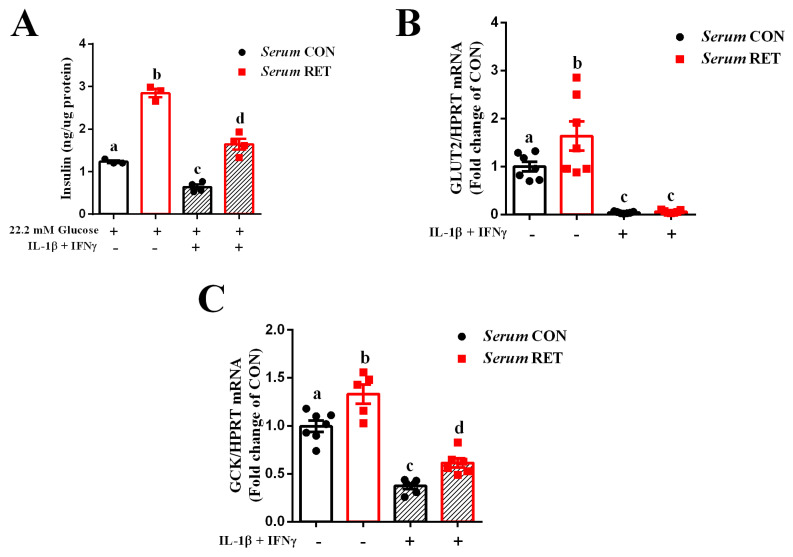
Serum from resistance-trained mice improves glucose sensing and preserves insulin secretion in INS-1E cells exposed to pro-inflammatory cytokines. INS-1E cells were incubated with conditioned medium containing 10% of serum from control or trained healthy mice for 24 h, followed by exposure to IL-1β plus IFN-γ for 24 h. Insulin secretion from INS-1E cells exposed to 22.2 mM glucose (n = 3–4) (**A**). Real-time PCR assay of GLUT2 (**B**) and glucokinase (GCK) (**C**) mRNA levels (n = 5–7) in INS-1E cells from different treatments, as indicated in the graph. The relative expression of mRNAs was determined after normalization with HPRT. Data are the mean ± SEM. Letters shared in common between groups indicate no significant difference. Different letters indicate statistical difference between groups, *p* ≤ 0.05 (one-way ANOVA).

Firstly, we investigated if resistance training would be able to preserve insulin secretion in beta cells exposed to an in vitro model of type 1 diabetes (T1D), and if such an effect could be mediated by exercise-induced factors released in the bloodstream. For this, we used a rat pancreatic beta cell line, called INS-1E, incubated with a medium containing 10% of serum from control (CON) or trained (RET) healthy mice for 24 h, followed by exposure to interleukin-1β (IL-1β) plus interferon-γ (IFN-γ) for 24 h. Under normal conditions, we observed that INS-1E cells incubated with trained serum secrete more insulin than cells incubated with control serum, in response to a stimulatory glucose concentration (22.2 mM). Moreover, when cells pretreated with control serum were exposed to IL-1β plus IFN-γ, insulin secretion was impaired. However, cells pretreated with trained serum and exposed to cytokines preserved the secretory function (Figure 1A).

Next, we assessed the expression of genes related to glucose metabolism in beta cells, such as glucose transporter 2 (GLUT2) and glucokinase (GCK). The treatment with trained serum increased the expression of both genes in INS-1E cells under normal conditions (Figure 1B,C). These outcomes suggest that resistance exercise may increase the ability of beta cells to uptake and metabolize glucose, leading to improved insulin secretion. Nonetheless, exposure to pro-inflammatory cytokines drastically reduced GLUT2 expression in INS-1E cells treated with both control and trained serum (Figure 1B). Moreover, GCK expression was impaired in cells cultured in a medium containing serum from control mice and exposed to cytokines. In contrast, treatment with trained serum preserved the expression of GCK (Figure 1C). Thus, resistance training may preserve glucose sensing in beta cells in the face of harmful conditions, contributing to maintenance of insulin secretion.

### 2.2. Serum from Resistance-Trained Mice Protects INS-1E Cells from Cytokine-Induced Apoptosis

**Figure 2:**
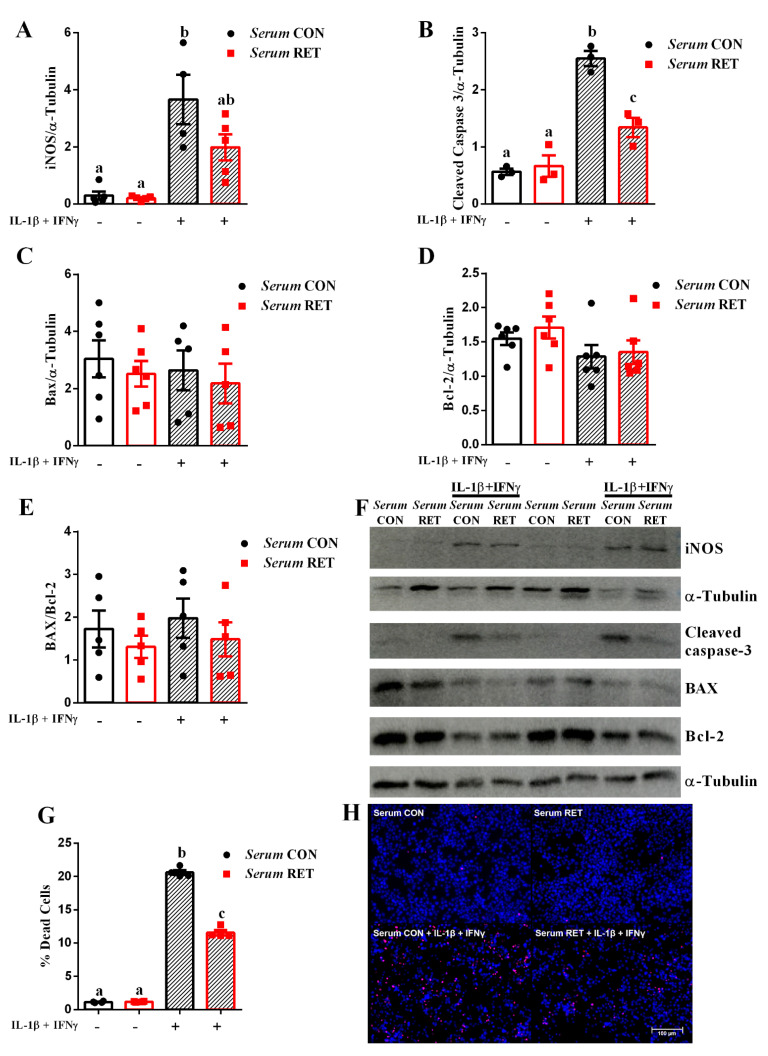
Serum from resistance-trained mice protects INS-1E cells from cytokine-induced apoptosis. INS-1E cells were incubated with conditioned medium containing 10% of serum from control or trained healthy mice for 24 h, followed by exposure to IL-1β plus IFN-γ for 24 h. Protein expression of iNOS (**A**), cleaved caspase-3 (**B**), BAX (**C**), and Bcl-2 (**D**) normalized by α-tubulin in INS-1E cells from different treatments as indicated in the graph (n = 3–6). BAX and Bcl-2 ratio is shown (**E**) (n = 5). Representative blots (**F**). Cell apoptosis was measured by HO and PI staining (n = 4) (**G**). Representative images from HO and PI stained cells (**H**). Data are the mean ± SEM. Letters shared in common between groups indicate no significant difference. Different letters indicate statistical difference between groups, *p* ≤ 0.05 (one-way ANOVA).

We also measured the expression of pro- and anti-apoptotic proteins, as well as apoptosis rate, and observed that in normal conditions there was no difference between pretreatment with control or trained serum. However, when cells were exposed to IL-1β plus IFN-γ, there was an increase in nitric oxide synthase (iNOS) (Figure 2A,F) and cleaved caspase-3 (Figure 2B,F) protein content in INS-1E cells pretreated with control serum, whereas in cells pretreated with trained serum, this effect was reduced. In addition, we evaluated the pro- and anti-apoptotic proteins B-cell lymphoma 2-associated X (BAX) and B-cell lymphoma 2 (Bcl-2), and there was no difference between groups regarding the content of these proteins, as well as their ratio (Figure 2C–F).

Finally, exposure to IL-1β plus IFN-γ increased beta cell apoptosis in both groups. However, INS-1E cells cultured in medium containing serum from trained mice were less affected by cytokine-induced apoptosis (Figure 2G,H). These data suggest that when beta cells are exposed to a pro-inflammatory environment (similar to the scenario observed in T1D), humoral factors induced by resistance exercise are able to protect these cells from injury and apoptosis.

### 2.3. Resistance Training Induces Adaptation and Alterations in Body Composition in Type 1 Diabetic Mice

**Figure 3:**
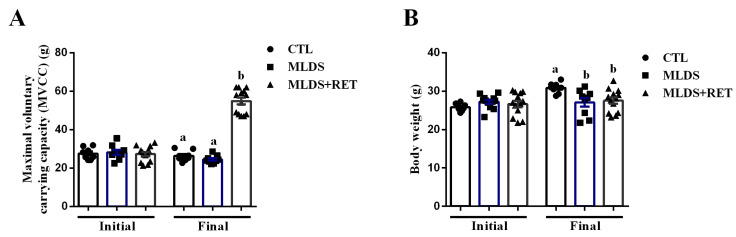
Resistance exercise increases maximal voluntary carrying capacity in diabetic trained mice. Initial and final maximal voluntary carrying capacity (MVCC) of CTL (n = 8), MLDS (n = 9), and MLDS + RET (n = 13) (**A**). Initial and final body weight (**B**) of CTL (n = 8), MLDS (n = 9), and MLDS + RET (n = 13). Data are the mean ± SEM. The absence of letters or letters shared in common between groups indicate no significant difference. Different letters indicate statistical difference between groups, *p* ≤ 0.05 (one-way ANOVA).

Based on our experiments in vitro, we investigated if resistance training improves glycemic control and beta cell mass and function in a mice model of type 1 diabetes (T1D). For this, we used C57Bl/6 mice, turned diabetic by multiple low doses of streptozotocin (MLDS). Then, these mice were submitted, or not, to resistance training for a 10-week period. Thus, we evaluated the maximal voluntary carrying capacity (MVCC) to assess the efficiency of our training program. As expected, after 10 weeks of training, diabetic-trained mice (MLDS + RET) presented higher MVCC, compared with both control (CTL) and diabetic sedentary (MLDS) mice (Figure 3A). Additionally, the training protocol increased the performance as judged by the ability of diabetic-trained mice to carry progressively heavier loads during the course of the 10 weeks of training (Figure S1).

Moreover, MLDS and MLDS + RET mice displayed a similar body weight, which was lower than CTL mice (Figure 3B). The MLDS group also presented lower gastrocnemius and soleus weight, as well as perigonadal and retroperitoneal fat pads compared to the CTL group. Nevertheless, gastrocnemius weight from MLDS + RET mice was similar to CTL, and soleus weight was even higher than that observed in the CTL mice. In addition, MLDS + RET mice presented a higher perigonadal fat pad compared to MLDS mice, while there was no difference between the diabetic groups concerning retroperitoneal fat pad weight (Table 1).

### 2.4. Resistance Exercise Training Modulates Glucose Metabolism in a Mice Model of Type 1 Diabetes

**Figure 4:**
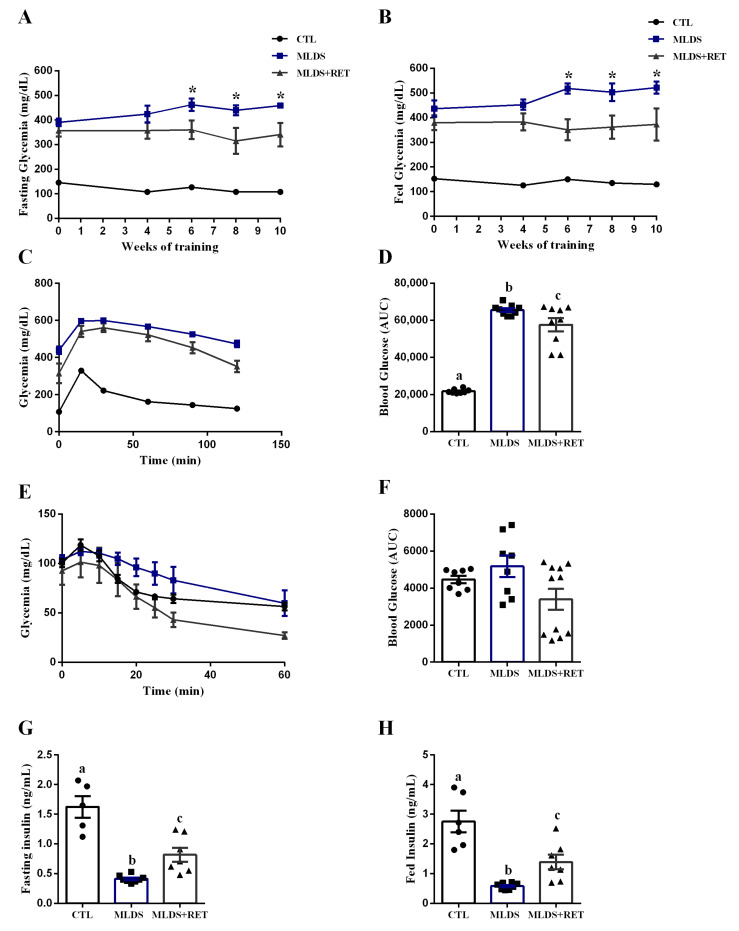
Resistance training improves glucose tolerance, reduces glycemia, and increases insulinemia in diabetic mice. Fasting (**A**) and fed (**B**) glycemia at weeks 0, 4, 6, 8, and 10 of the training period; CTL (n = 7–8), MLDS (n = 8–9), and MLDS + RET (n = 9–13). Blood glucose (**C**) and area under the curve (AUC) (**D**) of total blood glucose concentration of CTL (n = 8), MLDS (n = 9), and MLDS + RET (n = 9) during ipGTT. Blood glucose (**E**) and area under the curve (AUC) (**F**) of total blood glucose concentration of CTL (n = 8), MLDS (n = 8), and MLDS + RET (n = 11) during ipITT. Plasma insulin of CTL (n = 5–6), MLDS (n = 8), and MLDS + RET (n = 7) groups in fasting (**G**) and fed (**H**) states. Data are the mean ± SEM. Letters shared in common between groups indicate no significant difference. Different letters and (*) indicate statistical difference between groups, *p* ≤ 0.05 (one-way ANOVA or Kruskal–Wallis).

To access the effects of resistance exercise training on glucose homeostasis, we evaluated fasting and fed glycemia, and performed glucose and insulin tolerance tests (ipGTT and ipITT). Fasting (Figure 4A) and fed (Figure 4B) glycemia were higher in MLDS mice compared to CTL in all weeks evaluated. Nevertheless, from the sixth week of training until the end of the experimental period, MLDS + RET mice presented lower glycemia compared to MLDS mice, in both states.

Furthermore, glucose tolerance was impaired in MLDS mice compared to CTL, whereas diabetic-trained mice displayed improved glucose tolerance (Figure 4C), as determined by the lower area under the curve (AUC) of blood glucose during ipGTT compared to MLDS mice (Figure 4D). In addition, there was no difference between the groups regarding insulin sensitivity (Figure 4E,F). As expected, plasma insulin levels in fasting and fed states (Figure 4G,H) were significantly lower in the MLDS group compared to CTL. However, diabetic mice submitted to resistance training showed higher plasma insulin levels (both in fasting and fed states) than diabetic sedentary mice, which may contribute to the reduced glycemia, as well as improved glucose tolerance, observed in this group.

### 2.5. Resistance Training Increases Beta Cell Mass in Type 1 Diabetic Mice

**Figure 5:**
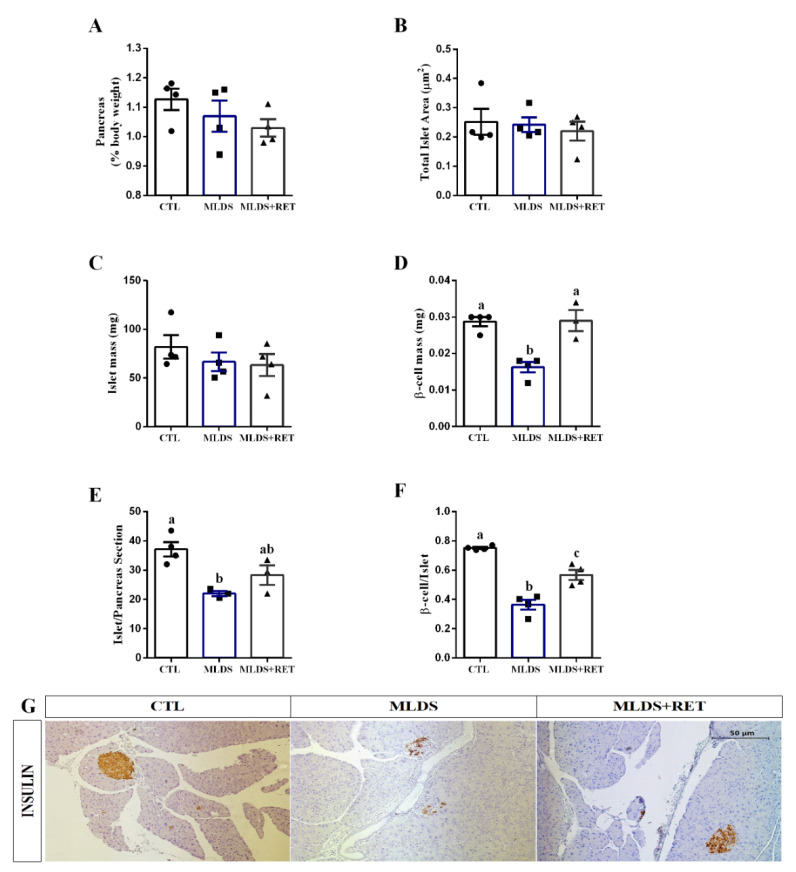
Resistance training increases beta cell mass in type 1 diabetic mice. Pancreas weight (% body weight) (**A**); total islet area (µm^2^) (**B**); islets mass (mg) (**C**); beta cell mass (mg) (**D**); islet/pancreas section ratio (**E**); and beta cell/islet ratio (**F**) of CTL (n = 4), MLDS (n = 3–4), and MLDS + RET (n = 3–4) mice. Representative images of pancreas sections stained for insulin (**G**). Data are the mean ± SEM. Letters shared in common between groups indicate no significant difference. Different letters indicate statistical difference between groups, *p* ≤ 0.05 (one-way ANOVA or Kruskal–Wallis).

Since diabetic resistance-trained mice display increased insulin levels, we further investigated if this effect could be linked with alterations in beta cell and islet morphology. We observed that pancreas weight (Figure 5A), total islet area (Figure 5B), and islet mass (Figure 5C) remained similar in all groups. However, beta cell mass (Figure 5D), islet/pancreas section (Figure 5E), and beta cell number per islet (Figure 5F) were reduced by 43%, 40%, and 51%, respectively, in the MLDS group compared to the CTL group. Interestingly, resistance training restored these parameters in MLDS + RET mice. Representative figures of the histological pancreatic sections, stained for insulin, are shown in Figure 5G.

## 3. Discussion

Here, we provide evidence that resistance exercise training improves glucose sensing, and protects beta cells from injury and apoptosis in an in vitro model of diabetes. In addition, streptozotocin-induced diabetic mice present improved glucose homeostasis and higher beta cell mass when submitted to resistance training. Based on these findings, we propose that resistance training may induce the release of humoral factors which can help beta cells become more resistant to harmful conditions and improve their response to a glucose stimulus.

It is known that the serum from resistance-trained mice preserves insulin secretion in INS-1E cells when they are exposed to a chemical endoplasmic reticulum (ER) stressor. This protective role is linked with increased expression of the insulin 2 (Ins2) gene and reduction of ER stress markers [21]. Similarly, we observed that insulin secretion in INS-1E cells was impaired when they were exposed to pro-inflammatory cytokines, whereas pre-treatment with the serum from resistance-trained mice preserved the secretory function of these cells (Figure 1A).

The beneficial effect of trained serum in INS-1E cells exposed to pro-inflammatory cytokines could be linked with alterations in glucose metabolism. Pancreatic endocrine cells are able to uptake glucose through members of the facilitative glucose transporter (GLUT) family (SLC2) and the sodium-glucose cotransporter (SGLT) family (SLC5) [23,24]. Inside the beta cell, glucose is phosphorylated by glucokinase (GCK) and metabolized to pyruvate. Mitochondrial oxidation of pyruvate results in an increase of intracellular ATP, triggering a series of events that leads to exocytosis of insulin containing vesicles [23,24,25]. Thus, alterations in the expression of glucose transporters, as well as GCK, lead to impaired glucose-stimulated insulin secretion (GSIS). In this context, different diabetic models present reduced expression of GLUT2; which is the main glucose transporter on beta cells in rodents [24,26,27,28,29,30,31]. In addition, studies conducted using endurance exercise protocols have shown improvement of glucose sensing in mice models due to increased GLUT2 and GCK expression [11,12,15,32,33]. Here, we observed similar outcomes, since resistance-trained serum increased GLUT2 and GCK expression in INS-1E cells under normal conditions. Glucose phosphorylation by GCK is the rate-limiting factor for glucose utilization and GSIS in rodent beta cells [23], and this could explain the increase in insulin secretion observed in cells treated with resistance-trained serum. In contrast, pro-inflammatory cytokines impaired both GLUT2 and GCK expression, regardless of the type of serum used prior to cytokine exposure. The treatment with trained serum did not prevent cytokine-induced impairment on GLUT2 expression; however, it preserved the expression of GCK (Figure 1B,C). These findings suggest that resistance training is able to improve insulin secretion by increasing the capacity of beta cells to uptake and metabolize glucose, even in the face of inflammatory conditions.

Furthermore, we observed that exposure to IL-1β plus IFN-γ increased the expression of iNOS and cleaved caspase-3, as well as the apoptosis rate in INS-1E cells treated with control serum (Figure 2A,B,G). Indeed, pro-inflammatory cytokines (e.g., IL-1β, TNF-α, and IFN-γ) are involved in beta cell apoptosis. These cytokines activate two different pathways: signal transducer and activator of transcription (STAT)-1, and nuclear factor kappa B (NF-κB), which induce several target genes related to apoptosis [8,34,35]. NF-κB induces expression of iNOS in beta cells, which catalyzes the generation of nitric oxide (NO). NO reacts with prosthetic groups present in transcription factors and DNA fragmentation, and inhibits enzymatic activity, leading to decreased glucose oxidation, oxygen consumption, ATP synthesis activity, and insulin synthesis. In addition, NO may induce cytochrome c release from the mitochondrial membrane, which leads to the execution of the apoptotic signal through executioner caspase activation [36].

Exercise increases the content and activity of antioxidants enzymes and reduces reactive oxygen species, leading to reduced apoptotic markers, such as cleaved caspase-3 and BAX in pancreatic islets in mice models [8,14,37]. In addition, the anti-apoptotic effects of exercise in different tissues are due to reduced activation of the NF-κB pathway, iNOS expression, and NO production [8,38,39,40,41]; this could explain the reduced expression of iNOS and cleaved caspase-3, and consequently the reduced apoptosis rate in INS-1E cells treated with trained serum (Figure 2A,B,G). We also evaluated the expression of BAX and Bcl-2; the former promotes apoptosis, while the second one inhibits mitochondrial apoptotic pathways [36]. However, there was no difference between groups regarding both the expression of these proteins and their ratio (Figure 2C–E), suggesting that this model does not involve the mitochondrial pathway of apoptosis. Similarly, serum from endurance-trained mice and humans protect rodent and human beta cells from cytokine and ER stressor-induced apoptosis, through an IL-6/STAT3 dependent pathway [8,9]. Exercise may also reduce beta cell apoptosis by regulating immunity. Evidence demonstrate that exercise reduces immune cell infiltration into the pancreas, and subsequently insulitis in non-obese diabetic (NOD) mice [42]. Additionally, moderate intensity exercise in NOD mice reduces inflammatory markers and islet infiltration, contributing to the reduction of apoptosis [13].

The in vivo experiments demonstrated that streptozotocin-induced diabetic mice present reduced body weight (Figure 3B), as well as altered body composition (Table 1) in comparison to their control littermates; similar to other studies using streptozotocin to induce diabetes [43,44,45,46]. The reduction in fat depots and muscle weight observed in MLDS mice could be linked with their lower plasma insulin levels (Figure 4G,H), since insulin is an anabolic hormone responsible for stimulating several pathways of synthesis and storage in skeletal muscle and adipose tissue [24,47]. Moreover, the same features are observed in T1D patients in initial stages of the disease [48,49,50]. Interestingly, diabetic-trained mice presented higher muscle mass and fat deposition (Table 1), which could be explained, at least in part, by the increased insulin levels in fasting and fed states (Figure 4G,H).

Resistance training increases insulin secretion in response to a glucose stimulus, in both isolated islets from healthy mice and INS-1E cells treated with trained serum [21]. Thus, we believe that the higher insulinemia in MLDS + RET mice may be due to an exercise-induced increase in insulin secretion, which contributed to reduce glycemia, as well as improve glucose tolerance in these mice (Figure 4A–D). Other studies also demonstrate that resistance exercise improves beta cell function and insulin sensitivity in diabetic individuals [51,52]. In our study, insulin sensitivity was not altered in MLDS mice (Figure 4E,F); rather, they only displayed a lack of insulin, and not resistance to the hormone. In addition, resistance training did not seem to impact this parameter.

Exercise stimulates the release of different molecules in the bloodstream, called exerkines, which exert their effects through endocrine, paracrine, and/or autocrine pathways. Several organs and tissues release these factors, including skeletal muscle (myokines) [53]. Moreover, some of these myokines act upon beta cell function and survival [54], such as: IL-6 [8], irisin [55], BnDF [56], GDF-15 [57], follistatin [58], angiogenin and osteoprotegerin [59]. Furthermore, the profile of secreted molecules differs from one type of muscle fiber to another [59].

In this context, the results with resistance exercise are opposite to the effects of endurance exercise, which increases sensitivity and reduces insulin secretion [8,37,60,61]. These discrepancies may be related to the activation of different muscle fibers in each modality of exercise, and consequently, the signaling pathways involved [62,63]. Along these lines, further analysis of the serum extracted from resistance-trained individuals are needed to identify which humoral factors are involved with resistance exercise-induced effects on beta cells, which is a limitation of our study. Another point to consider is that resistance exercise stimulates muscle hypertrophy [64,65,66]; thus, it may somehow induce the increase of insulin secretion in order to attend the anabolic demand imposed by this type of exercise.

Studies using endurance exercise protocols demonstrate that exercise increases beta cell mass through distinguished mechanisms, such as increased cell proliferation and reduced apoptosis [8,9,12,32,33,37,67,68,69]. Here, we observed that resistance training also modulates the morphology of the pancreatic islet by increasing the number of islets per pancreas section, beta cell mass, and beta cell number per islet in diabetic mice (Figure 5D–F). Further investigation is needed to address the mechanism underlying this effect; however, it could be due to reduced apoptosis in a way similar to what we observed in the experiments in vitro, along with exercise-induced cell proliferation.

In conclusion, this study brings novel findings which support the notion that resistance training stimulates some protective pathways on beta cells, probably through exercise-induced factors released in the bloodstream. Even though further investigation still has to be done to understand the mechanisms behind the effects of this type of exercise on pancreatic endocrine cells, this could lead to the discovery of molecules that could help diabetic individuals maintain their glycemic control, as well as their remaining beta cells.

## 4. Materials and Methods

### 4.1. Experimental Design

For in vitro experiments, a rat pancreatic beta cell line (INS-1E) was treated with serum from control or resistance-trained healthy mice and exposed (or not) to pro-inflammatory cytokines. Additionally, we used a type 1 diabetic mice model (induced by multiple low doses of streptozotocin—MLDS) which was submitted to 10 weeks of resistance exercise training.

### 4.2. Mice

All experiments were approved by the Animal Care Committee at UNICAMP (Protocol #5068-1). Additionally, the study was carried out in compliance with the ARRIVE guidelines. Male, eight-week-old C57Bl/6 mice were obtained from the breeding colony at UNICAMP and maintained at 22 ± 1 °C on a 12 h light–dark cycle. During the experimental period, mice had free access to water and chow diet. All mice were housed collectively (5 mice per cage). The healthy mice were divided into two groups: the control (CON) group, which remained sedentary throughout the experimental period, and the resistance exercise training (RET) group, which underwent resistance training during 10 weeks. At the end of the training program, mice were weighed and euthanized (by decapitation after inhalation of isoflurane) for blood collection. The muscles soleus and gastrocnemius were removed and weighed. All the experiments were performed 6 h after the exercise session (as previously described [21]). Information regarding body weight, muscles weight, and maximal voluntary carrying capacity (MVCC) of CON and RET mice is found in Table S1.

For experiments using the mice model of type 1 diabetes, mice were divided into two groups: (1) the control (CTL) group, and (2) multiple low doses of streptozotocin (MLDS) group. To induce type 1 diabetes, we used the multiple low doses protocol, which consists of an intraperitoneal (i.p.) injection of streptozotocin (40 mg/kg, dissolved in 0.5 M citrate buffer, pH 4.5) (Sigma-Aldrich, St. Louis, MO, USA) administered for 5 consecutive days. The same volume of citrate buffer was injected in the CTL group. Mice with fasting blood glucose levels ≥ 200 mg/dL 12 days after the last streptozotocin injection were considered diabetic [70,71] (Figure S2). Diabetic mice were randomly selected and divided into the two following groups: (1) the MLDS group, which remained sedentary throughout the experimental period, as well as the CTL group; and (2) the MLDS + resistance exercise training (MLDS + RET) group, which underwent resistance training during 10 weeks. All mice were weighed in the first and the last week of the experimental period. Moreover, fasting and fed glycemia was measured at weeks 0, 4, 6, 8, and 10 of the training period. At the end of the training program, mice were euthanized for blood collection by decapitation after inhalation of isoflurane. The muscles soleus and gastrocnemius, as well as perigonadal and retroperitoneal fat pads, were removed and weighed. All the experiments were performed 6 h after the exercise session.

### 4.3. Resistance Exercise Training Protocol

Resistance exercise training was performed according to the previously described protocol [21]. Briefly, mice were familiarized with the training apparatus (a 105 cm high ladder of iron feet and stainless steel steps; AVS PROJECTS, São Carlos, SP, Brazil) for 4 consecutive days. After this period, we determined the maximal voluntary carrying capacity (MVCC) of each mouse [21,72,73,74], before the beginning of the training program. Based on the MVCC test, the training sessions consisted of 8 climbs at 4 different loads (2 climbs with each load): 50%, 75%, 90%, and 100% of the mice’s MVCC, with a rest interval of 60 sec between climbs. The resistance training was performed 5 days per week with 2 days of rest, during a 10-week period. During this period, MVCC was determined once a week (at the last training session of each week) to set the appropriate load for each mouse. Sedentary mice were only exposed to the training ladder at weeks 0 (initial) and 10 (final). At these two time points, all sedentary mice were submitted to the adaptation protocol, and then they performed the MVCC test.

### 4.4. INS-1E Cell Culture and Treatment

The rat insulin-producing INS-1E cell line was obtained from Professor C. Wollheim (Centre Medical Universitaire, Geneva, Switzerland). The cells were cultured in RPMI 1640 medium (VITROCELL, Campinas, SP, Brazil) and supplemented with 5% v/v of fetal bovine serum (FBS; VITROCELL, Campinas, SP, Brazil), HEPES 10 mmol/l, sodium pyruvate 1 mmol/l and 2-mercaptoethanol 50 µmol/l with 11 mmol/l glucose; in a humidified atmosphere at 37 °C and 5% CO_2_. Cells were used at passages 60–70. INS-1E cells were seeded in 24-well, 48-well, or 96-well culture plates until 70–80% confluence. Then, cells were incubated with medium containing 10% of serum from control (CON) or trained (RET) healthy mice (without FBS) for 24 h. Next, the medium was replaced by fresh growth medium (with FBS) containing 10 U/mL recombinant human interleukin-1β (IL-1β) plus 100 U/mL recombinant rat interferon-γ (IFN-γ) for 24 h (in vitro T1D) [8]. Finally, the cells were washed with phosphate-buffered saline (PBS) and used for insulin secretion, real-time PCR assays, Western blot analysis, and apoptosis measurement (by HO-PI fluorescence quantification).

### 4.5. Insulin Secretion in INS-1E Cells

After the culture treatment period, INS-1E cells (seeded in 24-well culture plates) were incubated for 1 h at 37 °C in Krebs-bicarbonate buffer (KBB) without glucose. This solution was replaced with fresh KBB containing 22.2 mM glucose (stimulatory glucose concentration) for 1 h. At the end of the incubation period, supernatants were collected and stored at −20 °C until insulin was measured by an ELISA Kit (cat. #EZRMI-13K; Merck Millipore, Darmstadt, Germany). INS-1E cells were subsequently washed with PBS, lysed in 60 µL of urea/thiourea buffer (7 M urea, 2 M thiourea, 100 mM Tris pH 7.5, 10 mM sodium pyrophosphate, 100 mM sodium fluoride, 10 mM ethylenediaminetetraacetic acid (EDTA), 10 mM sodium orthovanadate, 2 mM phenylmethylsulfonyl fluoride (PMSF), 1% Triton X-100 and 0.1 mg/mL aprotinin, 4 °C), and stored at −20 °C until assayed for total protein measurement by Bradford [75]. Insulin secretion was normalized by total protein.

### 4.6. mRNA Isolation and Real-Time Quantitative PCR

The extraction of total RNA content of INS-1E cells (seeded in 48-well culture plates) was performed using TRIzol^®^ reagent (Thermo Fisher Scientific, Waltham, MA, USA), following phenol-chloroform RNA extraction, according to the manufacturer’s recommendations. Nanodrop (Nanodrop Thermo scientific, Wilmington, DE, USA) was used to measure RNA concentration. cDNA was prepared using 0.750 µg RNA and MultiScribe reverse transcriptase (Applied Biosystems, Foster City, CA, USA). For PCR reactions, we used SYBR-green master mix (Applied Biosystems, Foster City, CA, USA). The 7500 Fast Real-time PCR System (Applied Biosystems, Foster City, CA, USA) was used for quantification. The specificities of amplifications were verified by melting-curve analyses. The relative expression of mRNAs was determined after normalization with hypoxanthine-guanine phosphoribosyltransferase (HPRT), using the 2-ΔΔCt method [76]. Primer sequences used for real-time qPCR assays are described in Table S2.

### 4.7. Western Blot

For Western blot, 30 µg of the total protein for nitric oxide synthase (iNOS) (cat. 610328; BD Biosciences, San Jose, CA, USA), B-cell lymphoma 2-associated X (Bax) (cat. #2772; Cell Signaling Technology, Beverly, MA, USA), B-cell lymphoma 2 (Bcl-2) (cat. #2870; Cell Signaling Technology, Beverly, MA, USA), cleaved caspase-3 (cat. #9662; Cell Signaling Technology, Beverly, MA, USA), and the housekeeping α-tubulin (cat. T6074; Sigma-Aldrich, St. Louis, MO, USA), from the INS-1E cells, were resolved using 8% and 12% SDS-PAGE and electroblotted onto nitrocellulose membranes. Immunodetection was performed after 1 h of blocking with 5% bovine serum albumin (BSA), at room temperature (RT), and an overnight period with specific primary antibody incubation at 4 °C, followed by exposure to an appropriate secondary antibody. Protein bands were visualized using the Amersham Imager 600 (GE Healthcare Life Sciences, Buckinghamshire, UK), which detected chemiluminescence. The band intensities were quantified using ImageJ software (National Institutes of Health, Bethesda, MD, USA).

### 4.8. HO-PI Fluorescence Quantification

Apoptotic cells were evaluated using DNA-binding dyes Hoechst 33342 (HO; 1 mg/mL) and propidium iodide (PI; 1 mg/mL) (both Thermo Fisher Scientific; Waltham, MA, EUA; cat. #H3570 and #P3566, respectively). After serum and cytokines treatment, INS-1E cells (seeded in 96-well culture plates) were incubated with HO-PI for 15 min. Next, the cells were observed and photographed by fluorescence microscopy. The cells were counted using ImageJ software (National Institutes of Health, Bethesda, MD, USA), considering a minimum of 500 cells per image [77].

### 4.9. Intraperitoneal Glucose (ipGTT) and Insulin (ipITT) Tolerance Tests

On the eighth week of the training program, CTL, MLDS, and MLDS + RET mice were subjected to 6 h of fasting after the training session, to perform the ipGTT. The fasting blood glucose level was measured (time 0) by a glucometer (Accu-chek^®^, Roche, Basileia, Switzerland). After, the mice received an i.p. glucose dose of 2 g/kg, and glycemia was measured at 15, 30, 60, 90, and 120 min. Two days later, after the training session, mice were subjected to 6 h fasting for the ipITT, and the glycemia was measured (by a glucometer) before (time 0) and 5, 10, 15, 20, 25, 30, and 60 min after the i.p. administration of 1 U/kg insulin.

### 4.10. Plasma Insulin Measurement

For insulin measurements, blood samples from CTL, MLDS, and MLDS + RET mice were collected in fed and fasted states at the end of the training program. To obtain plasma samples, blood samples were centrifuged at 11,000 rpm, for 15 min, at 4 °C. To measure plasma insulin, a Mouse Insulin ELISA Kit (cat. #EZRMI-13K; Merck Millipore, Darmstadt, Germany) was used, and the assay was performed as indicated by the kit protocol.

### 4.11. Pancreas Morphometry and Immunohistochemistry

For immunohistochemistry, pancreas samples from 4 mice from each group (CTL, MLDS, and MLDS + RET) were weighed and immersed in 10% formalin fixative solution for 72 h at RT. Then, the tissues were dehydrated and embedded in Paraplast (Sigma Aldrich, St. Louis, MO, USA). Serial sections (5 μm thick and 250 μm apart from each other) were mounted onto silanized slides. After paraffin removal, sections were rehydrated and incubated with citrate buffer (pH 6.0) for 30 min at 95 °C for antigen retrieval. The sections were blocked with 5% BSA at RT and incubated with insulin primary antibody diluted at 1:10,000 (cat. ab181547; Abcam, Cambridge, UK) overnight at 4 °C. For the immunoperoxidase assay, subsequently, endogenous peroxidase activity was blocked with 0.3% solution of hydrogen peroxide and incubated with anti-rabbit IgG-HRP secondary antibody diluted at 1:500 (cat. #sc2004; Santa Cruz Biotechnology, Dallas, TX, USA) for 2 h. Insulin-positive cells were detected with 3.3′-diaminobenzidine (Sigma Aldrich, St. Louis, MO, USA) solution. All slides were counterstained with Ehrlich’s hematoxylin and mounted in coverslips using Entellan (Merck, Darmstadt, Germany) [78,79].

All islets present in the sections were covered systematically by capturing images with an inverted microscope (Eclipse Ti, Nikon, Tokyo, Japan) equipped with a digital camera (DS-U3, Nikon, Tokyo, Japan) and NIS Elements Basic Research (3.2 Software, Nikon, Tokyo, Japan), using a 10x objective. Pancreatic islets and beta cells were measured using Image J software (National Institutes of Health, Bethesda, MD, USA). The pancreas weight was obtained immediately after the euthanasia. After immunohistochemistry, the measurement of the circumference of each islet was denominated as the total area of the islet. Islet/pancreas section ratio was presented as the total amount of islets in each section analyzed. The islet and beta cell mass were calculated by multiplying the total islet area and beta cell area by the pancreas weight (mg), and the beta cell/islet ratio was obtained by the total beta cell area divided by the respective islet area [70,80].

### 4.12. Statistical Analysis

The data are presented as the mean ± standard error of the mean (SEM). The sample size (n) used for the statistical analysis of each group in the experiments is described in the figure legends. To evaluate data normality, we applied the Shapiro–Wilk test. When normal, we used the parametric Student’s *t*-test (to compare two groups) or a one-way ANOVA with Tukey’s post hoc test (to compare three groups); otherwise, the non-parametric Mann–Whitney test (to compare two groups) or Kruskal–Wallis with Dunn’s post hoc test (to compare three groups) was adopted. The difference between groups was considered statistically significant if *p* ≤ 0.05.

## Figures and Tables

**Table 1:** Final characterization of control (CTL n = 8), multiple low doses of streptozotocin (MLDS n = 8–9), and MLDS + resistance exercise training (MLDS + RET n = 12–13) mice. Letters shared in common between groups indicate no significant difference. Different letters indicate statistical difference between groups, *p* ≤ 0.05. Data are presented as the mean ± SEM (one-way ANOVA or Kruskal–Wallis).

	CTL	MLDS	MLDS + RET
Retroperitoneal fat pad (% body weight)	0.515 ± 0.08 ^a^	0.098 ± 0.02 ^b^	0.184 ± 0.03 ^b^
Perigonadal fat pad (% body weight)	1.414 ± 0.19 ^a^	0.210 ± 0.07 ^b^	0.595 ± 0.09 ^c^
Gastrocnemius (% body weight)	1.025 ± 0.03 ^a^	0.836 ± 0.03 ^b^	1.087 ± 0.01 ^a^
Soleus (% body weight)	0.041 ± 0.003 ^a^	0.028 ± 0.001 ^b^	0.054 ± 0.002 ^c^

## Data Availability

Data are contained within the article or Supplementary Material.

## Supplementary Materials

The following supporting information can be downloaded at: https://www.mdpi.com/article/10.3390/ijms23169427/s1.

## References

[1] Apaolaza P.S., Balcacean D., Zapardiel-Gonzalo J., Nelson G., Lenchik N., Akhbari P., Gerling I., Richardson S.J., Rodriguez-Calvo T., nPOD-Virus Group (2021). Islet expression of type I interferon response sensors is associated with immune infiltration and viral infection in type 1 diabetes. Sci. Adv..

[2] Bathina S., Das U.N. (2021). Resolvin D1 Decreases Severity of Streptozotocin-Induced Type 1 Diabetes Mellitus by Enhancing BDNF Levels, Reducing Oxidative Stress, and Suppressing Inflammation. Int. J. Mol. Sci..

[3] DiMeglio L.A., Evans-Molina C., Oram R.A. (2018). Type 1 diabetes. Lancet.

[4] Galgani M., Bruzzaniti S., Matarese G. (2020). Immunometabolism and autoimmunity. Curr. Opin. Immunol..

[5] Metsios G.S., Moe R.H., Kitas G.D. (2020). Exercise and inflammation. Best Pract. Res. Clin. Rheumatol..

[6] Fernández-Rodríguez R., Monedero-Carrasco S., Bizzozero-Peroni B., Garrido-Miguel M., Mesas A.E., Martínez-Vizcaíno V. (2022). Effectiveness of Resistance Exercise on Inflammatory Biomarkers in Patients with Type 2 Diabetes Mellitus: A Systematic Review with Meta-Analysis. Diabetes Metab. J..

[7] Nguyen T.P., Jacobs P.G., Castle J.R., Wilson L.M., Kuehl K., Branigan D., Gabo V., Guillot F., Riddell M.C., Haidar A. (2021). Separating insulin-mediated and non-insulin-mediated glucose uptake during and after aerobic exercise in type 1 diabetes. Am. J. Physiol. Endocrinol. Metab..

[8] Paula F.M., Leite N.C., Vanzela E.C., Kurauti M.A., Freitas-Dias R., Carneiro E.M., Boschero A.C., Zoppi C.C. (2015). Exercise increases pancreatic β-cell viability in a model of type 1 diabetes through IL-6 signaling. FASEB J..

[9] Paula F.M.M., Leite N.C., Borck P.C., Freitas-Dias R., Cnop M., Chacon-Mikahil M.P.T., Cavaglieri C.R., Marchetti P., Boschero A.C., Zoppi C.C. (2018). Exercise training protects human and rodent β cells against endoplasmic reticulum stress and apoptosis. FASEB J..

[10] Bacchi E., Negri C., Zanolin M.E., Milanese C., Faccioli N., Trombetta M., Zoppini G., Cevese A., Bonadonna R.C., Schena F. (2012). Metabolic effects of aerobic training and resistance training in type 2 diabetic subjects: A randomized controlled trial (the RAED2 study). Diabetes Care.

[11] Choi S.B., Jang J.S., Park S. (2005). Estrogen and exercise may enhance beta-cell function and mass via insulin receptor substrate 2 induction in ovariectomized diabetic rats. Endocrinology.

[12] Choi S.B., Jang J.S., Hong S.M., Jun D.W., Park S. (2006). Exercise and dexamethasone oppositely modulate beta-cell function and survival via independent pathways in 90% pancreatectomized rats. J. Endocrinol..

[13] Codella R., Lanzoni G., Zoso A., Caumo A., Montesano A., Terruzzi I.M., Ricordi C., Luzi L., Inverardi L. (2015). Moderate Intensity Training Impact on the Inflammatory Status and Glycemic Profiles in NOD Mice. J. Diabetes Res..

[14] Coskun O., Ocakci A., Bayraktaroglu T., Kanter M. (2004). Exercise training prevents and protects streptozotocin-induced oxidative stress and beta-cell damage in rat pancreas. Tohoku J. Exp. Med..

[15] Park S., Hong S.M., Lee J.E., Sung S.R. (2007). Exercise improves glucose homeostasis that has been impaired by a high-fat diet by potentiating pancreatic beta-cell function and mass through IRS2 in diabetic rats. J. Appl. Physiol..

[16] Yardley J.E., Kenny G.P., Perkins B.A., Riddell M.C., Malcolm J., Boulay P., Khandwala F., Sigal R.J. (2012). Effects of performing resistance exercise before versus after aerobic exercise on glycemia in type 1 diabetes. Diabetes Care.

[17] Yardley J.E., Kenny G.P., Perkins B.A., Riddell M.C., Balaa N., Malcolm J., Boulay P., Khandwala F., Sigal R.J. (2013). Resistance versus aerobic exercise: Acute effects on glycemia in type 1 diabetes. Diabetes Care.

[18] Álvarez C., Ramírez-Campillo R., Ramírez-Vélez R., Izquierdo M. (2017). Effects and prevalence of nonresponders after 12 weeks of high-intensity interval or resistance training in women with insulin resistance: A randomized trial. J. Appl. Physiol..

[19] Liu Y., Ye W., Chen Q., Zhang Y., Kuo C.H., Korivi M. (2019). Resistance Exercise Intensity is Correlated with Attenuation of HbA1c and Insulin in Patients with Type 2 Diabetes: A Systematic Review and Meta-Analysis. Int. J. Environ. Res. Public Health.

[20] Reddy R., Wittenberg A., Castle J.R., El Youssef J., Winters-Stone K., Gillingham M., Jacobs P.G. (2019). Effect of Aerobic and Resistance Exercise on Glycemic Control in Adults with Type 1 Diabetes. Can. J. Diabetes.

[21] Bronczek G.A., Soares G.M., de Barros J.F., Vettorazzi J.F., Kurauti M.A., Marconato-Júnior E., Zangerolamo L., Marmentini C., Boschero A.C., Costa-Júnior J.M. (2021). Resistance exercise training improves glucose homeostasis by enhancing insulin secretion in C57BL/6 mice. Sci. Rep..

[22] Wolkowicz K.L., Aiello E.M., Vargas E., Teymourian H., Tehrani F., Wang J., Pinsker J.E., Doyle F.J., Patti M.E., Laffel L.M. (2021). A review of biomarkers in the context of type 1 diabetes: Biological sensing for enhanced glucose control. Bioeng. Transl. Med..

[23] Berger C., Zdzieblo D. (2020). Glucose transporters in pancreatic islets. Pflug. Arch..

[24] Rahman M.S., Hossain K.S., Das S., Kundu S., Adegoke E.O., Rahman M.A., Hannan M.A., Uddin M.J., Pang M.G. (2021). Role of Insulin in Health and Disease: An Update. Int. J. Mol. Sci..

[25] Shomali N., Mahmoudi J., Mahmoodpoor A., Zamiri R.E., Akbari M., Xu H., Shotorbani S.S. (2021). Harmful effects of high amounts of glucose on the immune system: An updated review. Biotechnol. Appl. Biochem..

[26] Ohneda M., Johnson J.H., Inman L.R., Chen L., Suzuki K., Goto Y., Alam T., Ravazzola M., Orci L., Unger R.H. (1993). GLUT2 expression and function in beta-cells of GK rats with NIDDM. Dissociation between reductions in glucose transport and glucose-stimulated insulin secretion. Diabetes.

[27] Ostenson C.G., Efendic S. (2007). Islet gene expression and function in type 2 diabetes; studies in the Goto-Kakizaki rat and humans. Diabetes Obes. Metab..

[28] Portha B., Giroix M.H., Serradas P., Gangnerau M.N., Movassat J., Rajas F., Bailbe D., Plachot C., Mithieux G., Marie J.C. (2001). Beta-cell function and viability in the spontaneously diabetic GK rat: Information from the GK/Par colony. Diabetes.

[29] Orci L., Unger R.H., Ravazzola M., Ogawa A., Komiya I., Baetens D., Lodish H.F., Thorens B. (1990). Reduced beta-cell glucose transporter in new onset diabetic BB rats. J. Clin. Invest.

[30] Thorens B., Weir G.C., Leahy J.L., Lodish H.F., Bonner-Weir S. (1990). Reduced expression of the liver/beta-cell glucose transporter isoform in glucose-insensitive pancreatic beta cells of diabetic rats. Proc. Natl. Acad. Sci. USA.

[31] Wang Z., Gleichmann H. (1998). GLUT2 in pancreatic islets: Crucial target molecule in diabetes induced with multiple low doses of streptozotocin in mice. Diabetes.

[32] Király M.A., Bates H.E., Kaniuk N.A., Yue J.T., Brumell J.H., Matthews S.G., Riddell M.C., Vranic M. (2008). Swim training prevents hyperglycemia in ZDF rats: Mechanisms involved in the partial maintenance of beta-cell function. Am. J. Physiol. Endocrinol. Metab..

[33] Park S., Hong S.M., Lee J.E., Sung S.R., Kim S.H. (2008). Chlorpromazine attenuates pancreatic beta-cell function and mass through IRS2 degradation, while exercise partially reverses the attenuation. J. Psychopharmacol..

[34] Eizirik D.L., Mandrup-Poulsen T. (2001). A choice of death—The signal-transduction of immune-mediated beta-cell apoptosis. Diabetologia.

[35] Rabinovitch A., Suarez-Pinzon W.L. (1998). Cytokines and their roles in pancreatic islet beta-cell destruction and insulin-dependent diabetes mellitus. Biochem. Pharmacol..

[36] Rojas J., Bermudez V., Palmar J., Martínez M.S., Olivar L.C., Nava M., Tomey D., Rojas M., Salazar J., Garicano C. (2018). Pancreatic Beta Cell Death: Novel Potential Mechanisms in Diabetes Therapy. J. Diabetes Res..

[37] Calegari V.C., Zoppi C.C., Rezende L.F., Silveira L.R., Carneiro E.M., Boschero A.C. (2011). Endurance training activates AMP-activated protein kinase, increases expression of uncoupling protein 2 and reduces insulin secretion from rat pancreatic islets. J. Endocrinol..

[38] Su S.H., Jen C.J., Chen H.I. (2011). NO signaling in exercise training-induced anti-apoptotic effects in human neutrophils. Biochem. Biophys. Res. Commun..

[39] Szalai Z., Szász A., Nagy I., Puskás L.G., Kupai K., Király A., Berkó A.M., Pósa A., Strifler G., Baráth Z. (2014). Anti-inflammatory effect of recreational exercise in TNBS-induced colitis in rats: Role of NOS/HO/MPO system. Oxid. Med. Cell. Longev..

[40] Chavoshan B., Fournier M., Lewis M.I., Porszasz J., Storer T.W., Da X., Rambod M., Casaburi R. (2012). Testosterone and resistance training effects on muscle nitric oxide synthase isoforms in COPD men. Respir. Med..

[41] Al-Jarrah M., Obaidat H., Bataineh Z., Walton L., Al-Khateeb A. (2013). Endurance exercise training protects against the upregulation of nitric oxide in the striatum of MPTP/probenecid mouse model of Parkinson’s disease. NeuroRehabilitation.

[42] Oharomari L.K., de Moraes C., Navarro A.M. (2017). Exercise Training but not Curcumin Supplementation Decreases Immune Cell Infiltration in the Pancreatic Islets of a Genetically Susceptible Model of Type 1 Diabetes. Sports Med. Open.

[43] Xu J., Liu T., Li Y., Yuan C., Ma H., Seeram N.P., Liu F., Mu Y., Huang X., Li L. (2018). Hypoglycemic and hypolipidemic effects of triterpenoid-enriched Jamun (Eugenia jambolana Lam.) fruit extract in streptozotocin-induced type 1 diabetic mice. Food Funct..

[44] Liu L., Du X., Zhang Z., Zhou J. (2018). Trigonelline inhibits caspase 3 to protect β cells apoptosis in streptozotocin-induced type 1 diabetic mice. Eur. J. Pharmacol..

[45] Jiang Y.P., Ye R.J., Yang J.M., Liu N., Zhang W.J., Ma L., Sun T., Niu J.G., Zheng P., Yu J.Q. (2020). Protective effects of Salidroside on spermatogenesis in streptozotocin induced type-1 diabetic male mice by inhibiting oxidative stress mediated blood-testis barrier damage. Chem. Biol. Interact..

[46] Carvalho A.L., DeMambro V.E., Guntur A.R., Le P., Nagano K., Baron R., de Paula F.J.A., Motyl K.J. (2018). High fat diet attenuates hyperglycemia, body composition changes, and bone loss in male streptozotocin-induced type 1 diabetic mice. J. Cell. Physiol..

[47] Kurauti M.A., Soares G.M., Marmentini C., Bronczek G.A., Branco R.C.S., Boschero A.C. (2021). Insulin and aging. Vitam. Horm..

[48] Chetan M.R., Thrower S.L., Narendran P. (2019). What is type 1 diabetes?. Medicine.

[49] Sala D., Zorzano A. (2015). Differential control of muscle mass in type 1 and type 2 diabetes mellitus. Cell. Mol. Life Sci..

[50] Gunawardana S.C., Piston D.W. (2012). Reversal of type 1 diabetes in mice by brown adipose tissue transplant. Diabetes.

[51] Croymans D.M., Paparisto E., Lee M.M., Brandt N., Le B.K., Lohan D., Lee C.C., Roberts C.K. (2013). Resistance training improves indices of muscle insulin sensitivity and β-cell function in overweight/obese, sedentary young men. J. Appl. Physiol..

[52] Tavakol L., Mahani M.N. (2019). Effects of resistance training on insulin resistance and pancreatic beta-cells function in male patients with type 2 diabetes. J. Phys. Act. Horm..

[53] Chow L.S., Gerszten R.E., Taylor J.M., Pedersen B.K., van Praag H., Trappe S., Febbraio M.A., Galis Z.S., Gao Y., Haus J.M. (2022). Exerkines in health, resilience and disease. Nat. Rev. Endocrinol..

[54] Barlow J.P., Solomon T.P. (2018). Do skeletal muscle-secreted factors influence the function of pancreatic β-cells?. Am. J. Physiol. Endocrinol. Metab..

[55] Natalicchio A., Marrano N., Biondi G., Spagnuolo R., Labarbuta R., Porreca I., Cignarelli A., Bugliani M., Marchetti P., Perrini S. (2017). The Myokine Irisin Is Released in Response to Saturated Fatty Acids and Promotes Pancreatic β-Cell Survival and Insulin Secretion. Diabetes.

[56] Fulgenzi G., Hong Z., Tomassoni-Ardori F., Barella L.F., Becker J., Barrick C., Swing D., Yanpallewar S., Croix B.S., Wess J. (2020). Novel metabolic role for BDNF in pancreatic β-cell insulin secretion. Nat. Commun..

[57] Nakayasu E.S., Syed F., Tersey S.A., Gritsenko M.A., Mitchell H.D., Chan C.Y., Dirice E., Turatsinze J.V., Cui Y., Kulkarni R.N. (2020). Comprehensive Proteomics Analysis of Stressed Human Islets Identifies GDF15 as a Target for Type 1 Diabetes Intervention. Cell Metab..

[58] Zhao C., Qiao C., Tang R.H., Jiang J., Li J., Martin C.B., Bulaklak K., Wang D.W., Xiao X. (2015). Overcoming Insulin Insufficiency by Forced Follistatin Expression in β-cells of db/db Mice. Mol. Ther..

[59] Rutti S., Dusaulcy R., Hansen J.S., Howald C., Dermitzakis E.T., Pedersen B.K., Pinget M., Plomgaard P., Bouzakri K. (2018). Angiogenin and Osteoprotegerin are type II muscle specific myokines protecting pancreatic beta-cells against proinflammatory cytokines. Sci. Rep..

[60] Kurauti M.A., Freitas-Dias R., Ferreira S.M., Vettorazzi J.F., Nardelli T.R., Araujo H.N., Santos G.J., Carneiro E.M., Boschero A.C., Rezende L.F. (2016). Acute Exercise Improves Insulin Clearance and Increases the Expression of Insulin-Degrading Enzyme in the Liver and Skeletal Muscle of Swiss Mice. PLoS ONE.

[61] Kurauti M.A., Costa-Júnior J.M., Ferreira S.M., Dos Santos G.J., Protzek A.O., Nardelli T.R., de Rezende L.F., Boschero A.C. (2016). Acute exercise restores insulin clearance in diet-induced obese mice. J. Endocrinol..

[62] Schnyder S., Handschin C. (2015). Skeletal muscle as an endocrine organ: PGC-1α, myokines and exercise. Bone.

[63] Schiaffino S., Reggiani C. (2011). Fiber types in mammalian skeletal muscles. Physiol. Rev..

[64] Biolo G., Maggi S.P., Williams B.D., Tipton K.D., Wolfe R.R. (1995). Increased rates of muscle protein turnover and amino acid transport after resistance exercise in humans. Am. J. Physiol..

[65] Chesley A., MacDougall J.D., Tarnopolsky M.A., Atkinson S.A., Smith K. (1992). Changes in human muscle protein synthesis after resistance exercise. J. Appl. Physiol..

[66] MacDougall J.D., Tarnopolsky M.A., Chesley A., Atkinson S.A. (1992). Changes in muscle protein synthesis following heavy resistance exercise in humans: A pilot study. Acta Physiol. Scand..

[67] Curran M., Drayson M.T., Andrews R.C., Zoppi C., Barlow J.P., Solomon T.P.J., Narendran P. (2020). The benefits of physical exercise for the health of the pancreatic β-cell: A review of the evidence. Exp. Physiol..

[68] Király M.A., Bates H.E., Yue J.T., Goche-Montes D., Fediuc S., Park E., Matthews S.G., Vranic M., Riddell M.C. (2007). Attenuation of type 2 diabetes mellitus in the male Zucker diabetic fatty rat: The effects of stress and non-volitional exercise. Metabolism.

[69] Li Y., Xiao J., Tian H., Pei Y., Lu Y., Han X., Liu Y., Zhong W., Sun B., Fang F. (2013). The DPP-4 inhibitor MK0626 and exercise protect islet function in early pre-diabetic kkay mice. Peptides.

[70] Bronczek G.A., Vettorazzi J.F., Soares G.M., Kurauti M.A., Santos C., Bonfim M.F., Carneiro E.M., Balbo S.L., Boschero A.C., Costa Júnior J.M. (2019). The Bile Acid TUDCA Improves Beta-Cell Mass and Reduces Insulin Degradation in Mice with Early-Stage of Type-1 Diabetes. Front. Physiol..

[71] Furman B.L. (2015). Streptozotocin-Induced Diabetic Models in Mice and Rats. Curr. Protoc. Pharmacol..

[72] Hornberger T.A., Farrar R.P. (2004). Physiological hypertrophy of the FHL muscle following 8 weeks of progressive resistance exercise in the rat. Can. J. Appl. Physiol..

[73] Pereira R.M., Rodrigues K.C.D.C., Anaruma C.P., Sant’Ana M.R., de Campos T.D.P., Gaspar R.S., Canciglieri R.D.S., de Melo D.G., Mekary R.A., da Silva A.S.R. (2019). Short-term strength training reduces gluconeogenesis and NAFLD in obese mice. J. Endocrinol..

[74] Lima Y.C., Kurauti M.A., da Fonseca Alves G., Ferezini J., Piovan S., Malta A., de Almeida F.L.A., Gomes R.M., de Freitas Mathias P.C., Milani P.G. (2019). Whey protein sweetened with. Nutr. Metab..

[75] Bradford M.M. (1976). A rapid and sensitive method for the quantitation of microgram quantities of protein utilizing the principle of protein-dye binding. Anal. Biochem..

[76] Soares G.M., Zangerolamo L., Costa-Júnior J.M., Vettorazzi J.F., Carneiro E.M., Saad S.T., Boschero A.C., Barbosa-Sampaio H.C. (2019). Whole-Body ARHGAP21-Deficiency Improves Energetic Homeostasis in Lean and Obese Mice. Front. Endocrinol..

[77] Mateus Gonçalves L., Vettorazzi J.F., Vanzela E.C., Figueiredo M.S., Batista T.M., Zoppi C.C., Boschero A.C., Carneiro E.M. (2019). Amino acid restriction increases β-cell death under challenging conditions. J. Cell. Physiol..

[78] Dos Santos C., Rafacho A., Ferreira S.M., Vettorazzi J.F., Dos Reis Araújo T., Mateus Gonçalves L., Ruhrmann S., Bacos K., Ling C., Boschero A.C. (2021). Excess of glucocorticoids during late gestation impairs the recovery of offspring’s β-cell function after a postnatal injury. FASEB J..

[79] Chung C.H., Hao E., Piran R., Keinan E., Levine F. (2010). Pancreatic β-cell neogenesis by direct conversion from mature α-cells. Stem Cells.

[80] Lubaczeuski C., Balbo S.L., Ribeiro R.A., Vettorazzi J.F., Santos-Silva J.C., Carneiro E.M., Bonfleur M.L. (2015). Vagotomy ameliorates islet morphofunction and body metabolic homeostasis in MSG-obese rats. Braz. J. Med. Biol. Res..

