# Supplementary material for: Resistance Training Improves Beta Cell Glucose Sensing and Survival in Diabetic Models
Source: Int J Mol Sci. 2022 Aug 21;23(16):9427. doi: 10.3390/ijms23169427 (PMC9409046; doi:10.3390/ijms23169427)
Supplement: Supplementary file 1 [file ijms-23-09427-s001.zip › ijms-1843236-supplementary.pdf]

Supplementary Material

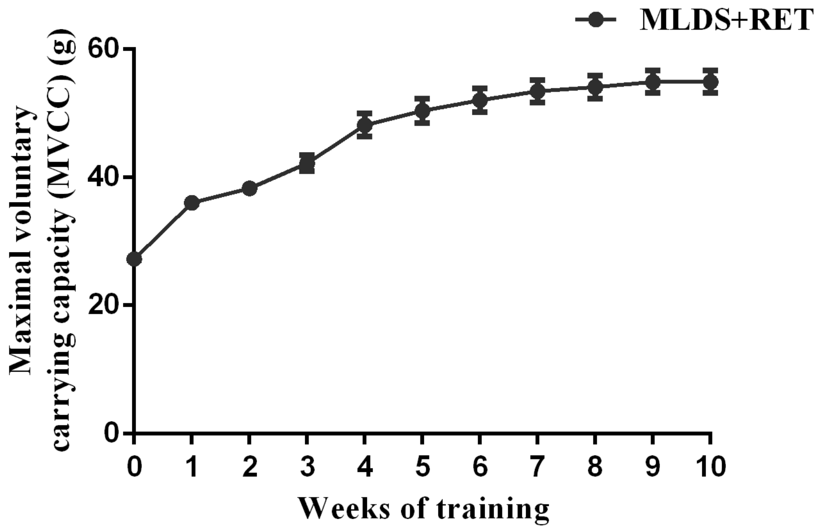

Figure S1. Maximal carrying load per week over the course of 10 weeks of the training program from MLDS+RET mice (n=13). Data are the mean ± SEM.

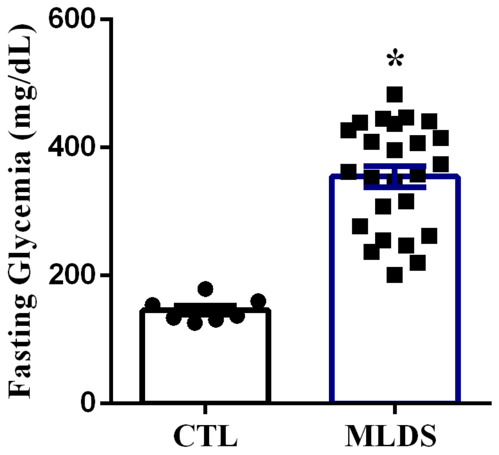

Figure S2. Fasting glycemia of CTL (n=7) and MLDS (n=25) mice, twelve days after the last streptozotocin administration. Data are the mean ± SEM. (*) Indicate statistical difference between groups, $P \leq 0.05$ (Student's T test).

Table S1. Final characterization of Control (CON n=6-7) and Resistance exercise training (RET n=6-7) mice. (*) Indicate statistic difference between groups. Data are presented as the mean ± SEM (Student's T test).

	CON	RET
**Maximal voluntary carrying capacity (MVCC) (g)**	42.63±2.01	68.34±1.60*
**Body weight (g)**	30.99±1.36	26.57±1.06*
**Gastrocnemius (% body weight)**	0.9157±0.02	1.010±0.01*
**Soleus (% body weight)**	0.1483±0.007	0.1950±0.007*

Table S2. Primer sequences for real-time qPCR assays.

Gene	Forward (5' – 3')	Reverse (3' – 5')
**GLUT2**	AGGTGACTGGGTCTCATTGGTG	CCTTCTTAACCAAGGCTGCGT
**GCK**	AGAAGCACCGACTGTGACTG	TGCTGAGCTGTGAGGAACTG
**HPRT**	TCCTCATGGACTGATTATGGACA	TAATCCAGCAGGTCAGCAAAGA

GLUT2: Glucose transporter 2; GCK: Glucokinase; HPRT: Hypoxanthine-guanine phosphoribosyltransferase.
